# Impact of extended fertilization on soil microbial diversity: a comparative full-length 16S rRNA amplicon analysis

**DOI:** 10.1186/s44342-026-00078-3

**Published:** 2026-10-13

**Authors:** Kazi Chamonara, Md. Habib Ullah Masum, Fahmida Ferdous Faria, Salma Chowdhury, Md Sadequr Rahman Khan, Md. Abul Kashem

**Affiliations:** 1 Department of Environmental Biotechnology, Faculty of Biotechnology and Genetic Engineering, Chattogram Veterinary and Animal Sciences University, Khulshi, Chattogram, 4225 Bangladesh; 2 Department of Genomics and Bioinformatics, Faculty of Biotechnology and Genetic Engineering, Chattogram Veterinary and Animal Sciences University, Khulshi, Chattogram, 4225 Bangladesh; 3 Department of Soil Science, University of Chittagong, Chattogram, 4331 Bangladesh; 4 Department of Industrial Biotechnology, Faculty of Biotechnology and Genetic Engineering, Chattogram Veterinary and Animal Sciences University, Khulshi, Chattogram, 4225 Bangladesh; 5 Department of Marine Bioresource Science, Faculty of Fisheries, Chattogram Veterinary and Animal Sciences University, Khulshi, Chattogram, 4225 Bangladesh

**Keywords:** Nanopore sequencing, Heavy metal analysis, Soil bacterial community, Long-term fertilization, Bangladesh

## Abstract

**Background:**

Soil bacterial communities are essential for regulating soil fertility, nutrient cycling, and agricultural sustainability. However, their responses to different fertilization regimes, especially in intensively managed agroecosystems, remain inadequately characterized in Chattogram.

**Methods:**

This study assessed the effects of prolong applications of chemical and organic fertilizers on soil bacterial community structure using culture-independent techniques (Oxford Nanopore long-read 16S rRNA gene sequencing).

**Result:**

In subsequent taxonomic composition analyses, beta diversity revealed significant differences in bacterial community composition among the fertilization regimes (*p* < 0.05), suggesting that long-term differences in fertilization practices may be associated with variation in bacterial community structure. The *R*^2^ value (0.84) indicates that the fertilization regime was associated with a substantial proportion of the observed variation in bacterial community composition. However, organic amendment was associated with significantly higher abundances of several bacterial taxa linked to plant growth promotion and nutrient cycling than chemical fertilization. Among the predominant taxa, *Bacillus* was represented by 8.44 ± 1.255% under chemical fertilization and 14.77 ± 0.790% under organic amendment, while Gemmataceae accounted for 7.65 ± 0.689% and 11.60 ± 0.441%, respectively. A similar upward trend was observed among the less abundant taxa, with Xanthobacteraceae comprising 2.88 ± 0.197% and 4.15 ± 0.204%, and *Tumebacillus* 1.35 ± 0.171% and 2.73 ± 0.183%, under chemical fertilization and organic amendment, respectively (FDR < 0.001). *Bradyrhizobium* and *Paenibacillus* showed relatively similar abundances between the two fertilization treatments. *Bradyrhizobium* accounted for 3.33 ± 0.240% under chemical fertilization and 2.90 ± 0.216% under organic amendment (FDR = 0.055), whereas *Paenibacillus* represented 2.02 ± 0.173% and 2.50 ± 0.155%, respectively (FDR < 0.001). METAGENassist predictions and multivariate functional profiling suggest that organic amendments may enhance microbial capacity for the decomposition of organic matter and nutrient turnover.

**Conclusion:**

These current findings suggest that the effects of fertilization on soil bacterial communities may extend beyond overall richness and may involve changes in community structure and relative abundance. However, the effects of fertilization on soil fertility and long-term agricultural sustainability should be assessed in the context of broader soil attributes and microbial community features.

**Supplementary Information:**

The online version contains supplementary material available at https://doi.org/10.1186/s44342-026-00078-3.

## Introduction

Soil contains one of the most complex microbial ecosystems, which plays a central role in regulating key soil functions, including nutrient cycling, organic matter turnover, and carbon sequestration [1, 2]. These ecosystem services directly influence soil fertility and crop productivity [3, 4]. The functional diversity of soil bacterial communities enhances the resilience of agricultural systems by sustaining essential ecosystem processes. This diversity buffers agroecosystems against environmental fluctuations [5, 6]. Empirical studies suggest that agricultural management practices that promote beneficial microbial diversity, including the use of organic amendments, biofertilizers, and integrated fertilization strategies, can enhance soil microbial activity and community functionality [7, 8]. These enhancements increase nutrient availability and improve soil physicochemical properties, thereby supporting increased crop growth and productivity [7]. Changes in microbial community structure are also linked to shifts in soil nutrients and plant productivity [9, 10]. In this context, beneficial bacteria, particularly plant growth-promoting bacteria (PGPB), have broader implications for microbial ecology in agricultural systems [11]. These PGPB promote plant growth and development via direct or indirect mechanisms such as biological nitrogen-fixing, phosphate solubilization, siderophore production, phytohormone synthesis, and stress response modulation [11]. Also, PGPB may indirectly enhance plant development by reducing phytopathogens via the generation of antimicrobial metabolites, contending for resources and ecological niches, and systemic resistance in plants [12, 13]. Among PGPB, *Bacillus*, *Bradyrhizobium*, and *Paenibacillus* are well-documented bacterial genera that have been widely studied for their association with plant growth promotion and their ecological significance in agricultural soils [14–17].

Soil microbial communities are strongly influenced by the combined effects of physicochemical properties, nutrient availability, and heavy metal concentrations [18, 19]. Microbial growth, substrate availability, nutrient cycling, and habitat conditions are all influenced by soil pH and organic matter, which in turn affects microbial abundance and community composition [20–22]. Among these variables, pH is important since it affects nutrition, metal availability, and microbial physiology [18, 21]. Although nutrient enrichment may benefit certain microbial species, available N, P, K, and organic carbon offer vital resources for microbial growth and metabolism [23, 24]. In contrast, elevated concentrations of heavy metals such as Cd, Pb, Cr, Cu, Ni, and Zn can impose selective pressure by disrupting enzymatic activity, cellular metabolism, and membrane integrity, potentially reducing microbial diversity and altering community structure [18, 25]. Therefore, rather than being influenced by a single factor, microbial community structure reflects the combined impacts of soil chemistry, nutritional status, and metal stress [26].

Since 1961, synthetic fertilizer usage has increased more than tenfold, reaching approximately 200 million metric tons annually [27]. Bangladesh exemplifies this trend in agricultural intensification, with fertilizer use rising from 0.9 million tons in 1980 to over 4.5 million tons in recent years [28, 29]. The use of chemical fertilizers can reduce soil bacterial diversity, potentially weakening soil ecosystem resilience to climate-related stresses such as drought, flooding, salinity, and temperature extremes in South Asia [1]. In densely populated countries like as Bangladesh, negative externalities, including water eutrophication and groundwater nitrate contamination, present significant risks to long-term agricultural sustainability [30]. Prolonged use of nitrogen fertilizers reduces bacterial diversity, simplifies microbial networks, and promotes copiotrophic taxa, while suppressing oligotrophic bacteria that are essential for organic matter decomposition and nitrogen-fixing [31, 32]. In rice paddies, synthetic fertilizers decrease populations of beneficial nitrogen-fixing bacteria such as *Bradyrhizobium* and *Azospirillum* [33, 34]. Conversely, organic fertilization strategies are widely reported to promote bacterial diversity, increase microbial biomass, and improve functional stability in soil ecosystems [34, 35]. The organic amendments provide diverse carbon substrates that support a wider range of bacterial taxa, increase soil organic carbon, and stimulate beneficial microbial activity [36]. Such management increases bacterial richness by 10 to 20%, with particularly strong effects on *Acidobacteria*, *Verrucomicrobia*, and *Actinobacteria*, and also improves soil enzymatic activities related to nutrient cycling [37].

Although the ecological significance of the soil microbiome is well recognized, significant knowledge gaps persist concerning the effects of prolong chemical fertilization on soil bacterial community structure in agricultural area of Chattogram region. While many studies have used culture-dependent methods to evaluate microbial communities, a comprehensive understanding of fertilization-driven microbial dynamics in Bangladeshi soils remains insufficient [38–41]. Traditional culture-dependent methods detect less than 1% of soil bacteria due to cultivation limitations [42]. In contrast, high-throughput 16S rRNA gene amplicon sequencing has transformed soil bacterial ecology by enabling comprehensive profiling of entire communities directly from environmental DNA [43]. Therefore, a comprehensive investigation is required to compare soil bacterial communities under prolong integrated organic and conventional chemical fertilization systems in this agricultural area using culture-independent approach.

This study characterizes soil bacterial communities under chemical and organic fertilization using culture-independent approach such as Oxford Nanopore long-read 16S rRNA sequencing. By comparing community composition, diversity, and predominant taxa, we aimed to elucidate bacterial shifts associated with long-term fertilization. This study represents the first application of this sequencing strategy to investigate prolong fertilization-driven changes in bacterial communities in agricultural soils in Chattogram region.

## Methods

### Sample collection and processing

Soil samples were collected in December 2025 from two fertilization regimes: chemical fertilizer–treated soils (CF1–CF6) and organically fertilized soils (OF1–OF6). The two independent fertilization regimes were established in separate plots that have been long-term (~20 years) maintained under consistent field management by the Department of Soil Science, University of Chittagong, Bangladesh (Chattogram, latitude: 22°24 N, longitude: 91°50 E). In the chemical fertilization regime, urea [CO(NH_2_)_2_], triple superphosphate (TSP) [Ca(H_2_PO_4_)_2_·H_2_O], and muriate of potash (MOP; KCl) were used as the major sources of nitrogen (N), phosphorus (P), and potassium (K), respectively. In the organic fertilization regime, cattle manure was used as the organic fertilizer. Six independent plots were sampled for each treatment. Within each plot, multiple subsamples were randomly collected and pooled to generate a single composite sample. All samples were obtained from crop fields within the same agricultural area, where the respective fertilization regimes have been maintained as long-term management practices. Standard soil sampling procedures were followed: surface litter was removed, and soil was collected from topsoil (a depth of 0–20 cm) at approximately 50 m intervals using sterile stainless-steel spatulas [44]. Samples were randomly collected within the crop fields and subsequently pooled to form composite samples. The samples were stored in cool box and transported at 4 °C to the Central Genomics Laboratory, Department of Genomics and Bioinformatics, Chattogram Veterinary and Animal Sciences University (CVASU). Upon arrival, soil samples were stored at −80 °C until DNA extraction and microbial profiling.

### Physicochemical profiling and heavy metal assessment

Macro- and micronutrient concentrations of the soil samples were determined using standard analytical protocols established by Soil Resource Development Institute (SDRI), Bangladesh. Available phosphorus (P) was measured using the method described by Bray et. al. (1945) [45], and boron (B) was quantified with the azomethine-H colorimetric method [46]. Sulfur (S) content was determined using the acid seed digestion method [47]. Exchangeable potassium (K), calcium (Ca), and magnesium (Mg) were extracted with the ammonium acetate method [48]. Besides, total nitrogen (N) was determined using the Kjeldahl digestion method [49]. Bioavailable fractions of copper (Cu), iron (Fe), manganese (Mn), zinc (Zn), cadmium (Cd), nickel (Ni), lead (Pb), and chromium (Cr) were extracted using the diethylenetriaminepentaacetic acid (DTPA) method using Double Beam AAS Spectroscopy AA2210 [50]. Electrical conductivity (EC) was measured with an EC meter (Hanna Pocket Conductivity Tester HI-98303, DiST-3). Soil pH was assessed by the glass electrode method using a soil–water suspension at a 1:2.5 (w/v) ratio [51]. Organic carbon (OC) content was quantified with a LECO C-200 carbon analyzer [52]. Soil organic matter (OM) was determined using the Walkley–Black wet oxidation method [53].

### DNA extraction and 16S rRNA gene sequencing

Genomic DNA was extracted from Soil samples associated with both chemical and organic fertilizers for 12 representative samples using the DNeasy PowerSoil Pro Kit (Cat. No. 47014), in accordance with the manufacturer’s guidelines. The purified DNA samples were stored at −80 °C for subsequent analyses. Amplification of the V1–V9 region of the 16S rRNA (forward primer: 27 F, reverse primer: 1492R) gene was performed using Q5 High-Fidelity 2X Master Mix, following the manufacturer’s instructions [54, 55]. Polymerase chain reaction (PCR) amplification was performed with an initial denaturation at 95 °C for 3 min, followed by 30 cycles consisting of 95 °C for 15 s, 60 °C for 15 s, and 72 °C for 60 s. A final extension was carried out at 72 °C for 10 min. The amplified DNA was purified using AMPure XP reagent (Beckman Coulter, Inc.) and quantified with the Qubit 4 fluorometer and Qubit™ 1X dsDNA High Sensitivity (HS) reagent (Thermo Fisher Scientific, USA). For end preparation and dA tailing, 200 ng of DNA was used, followed by barcode ligation with the Native Barcoding Kit 96 V14 (SQK-NBD114.96; Oxford Nanopore). Sequencing adaptors were ligated using the NEBNext® Quick Ligation Module (NEB, E6056), and the resulting DNA libraries were pooled [56]. The fully adapter-ligated and purified library was combined with 37.5 μL of sequencing buffer and 25.5 μL of loading beads, then loaded onto the R10 flow cell (FLO-MIN114; Oxford Nanopore Technologies) for sequencing on the MinION Mk1C. Data were collected using MINKNOW software version 1.11.5 (Oxford Nanopore Technologies) [57].

A bacterial DNA and nuclease-free water served as positive and negative controls, respectively. Subsequently, the DNA samples were barcoded and sequenced separately to evaluate sequencing performance and analyze possible contamination. Control data were separated from soil-sample reads by their unique barcodes and sequencing identities, and only soil-sample reads were used in downstream alpha diversity, beta diversity, relative abundance, and differential composition analyses.

### Bioinformatic analysis

MinION Mk1C sequencing data (FAST5 files) were basecalled using Guppy v6.3.2 (Oxford Nanopore Technologies) in high-accuracy mode, resulting in pass reads in FASTQ format with a mean quality score greater than 9. Adapter and barcode sequences were trimmed using Porechop v0.2.4 [58], and low-quality bases were filtered with Nanofilt (>Q15) [59]. Quality-trimmed reads were filtered by size to retain sequences of approximately 1600 base pairs, corresponding to the V1–V9 region, based on the size distribution of 16S rRNA gene sequences. Taxonomic assignment was conducted using the wf-16s workflow in Epi2me software (Oxford Nanopore Technologies) [60], with SILVA [61] as the reference database and Kraken2 as the classifier. All other parameters were set to their default values [62]. Functional annotation and multivariate statistical analyses of microbial communities were performed using METAGENassist [63]. Taxonomic profiles obtained from 16S rRNA gene sequencing were normalized and subsequently mapped to predicted microbial metabolic functions. Multivariate analyses, such as principal component analysis, were utilized to assess functional differences among the fertilization treatments.

### Statistical analysis

Statistical analyses, including calculation of the mean and standard deviation (SD) [64], one-way analysis of variance (ANOVA) [65], and Spearman’s rank correlation coefficient [66], were performed using R software (v 4.1.3). The Phyloseq (v4.2) [67], Vegan [68], ggplot2 [69], and microbiomeutilities packages for R (v4.1.3) [70] were utilized for downstream analyses, including alpha and beta diversity, microbial composition, differential abundance calculation, and statistical comparisons. Taxonomic features data were standardized using total sum scaling (TSS) [71], and the resulting normalized data were employed to assess alpha and beta diversity. Within-sample microbial diversity (alpha diversity) was evaluated by calculating observed species, Chao1 [72], ACE, Shannon [73], Simpson [74], and InvSimpson indices using the phyloseq and vegan R packages [68]. Differences in microbial diversity and abundance among groups were assessed with the Wilcoxon rank-sum test implemented in the microbiomeutilities R package. Between-sample diversity (beta diversity) was examined by visualizing variations among sample groups using principal coordinate analysis (PCoA) and non-metric multidimensional scaling (NMDS) based on the Bray–Curtis distance [75, 76]. Permutational multivariate analysis of variance (PERMANOVA) with 999 permutations was conducted to test for statistically significant differences between groups [77].

Differences in the relative abundance of bacterial taxa between organic- and chemical-fertilized soil samples were evaluated using a two-sided Wilcoxon rank-sum test [78]. To control for multiple hypothesis testing, *p *values were adjusted using the Benjamini–Hochberg false discovery rate (FDR) correction [79]. The relationships between soil physicochemical properties and the relative abundance of bacterial genera were assessed using Spearman’s rank correlation analysis [66]. Statistical significance was determined at an FDR-adjusted *p *value < 0.05.

## Results

### Physicochemical and heavy metal properties of soil samples

The comparative analysis of soil nutrient composition revealed significant differences between chemical and organic fertilizer treatments. Soils receiving chemical fertilizers for prolong time period contained higher concentrations of P and B (35.33 ± 2.16 and 0.48 ± 0.04, respectively) compared to those treated with organic fertilizers (7.5 ± 1.05 and 0.35 ± 0.04; *p* < 0.001). Conversely, S, K, and N contents were significantly greater in soils amended with organic fertilizers (8.0 ± 0.89, 0.09 ± 0.01, and 0.11 ± 0.01, respectively) than in those treated with chemical fertilizers (*p* < 0.001). Ca and Mg levels did not differ significantly between the two treatments (*p* > 0.05) (Table 1, Data S1).

**Table 1:** The nutrient composition in chemical and organic fertilizer amended soils

Nutrient composition	Soil treatment type		*p* value
	Chemical fertilizer (mean ± SD)	Organic fertilizer (mean ± SD)	
P (mg/kg)	35.33 ± 2.16	7.5 ± 1.05	<0.001
B (mg/kg)	0.48 ± 0.04	0.35 ± 0.04	<0.001
S (mg/kg)	5 ± 0.89	8 ± 0.89	<0.001
K (meq/100 g)	0.06 ± 0.01	0.09 ± 0.01	<0.001
Ca (meq/100 g)	3.6 ± 0.09	3.6 ± 0.09	1.00
Mg (meq/100 g)	3.43 ± 0.16	3.44 ± 0.15	0.93
N (%)	0.09 ± 0.01	0.11 ± 0.01	<0.001

Note: The *p* value < 0.05 implies a statistically significant difference

**Fig. 1:**
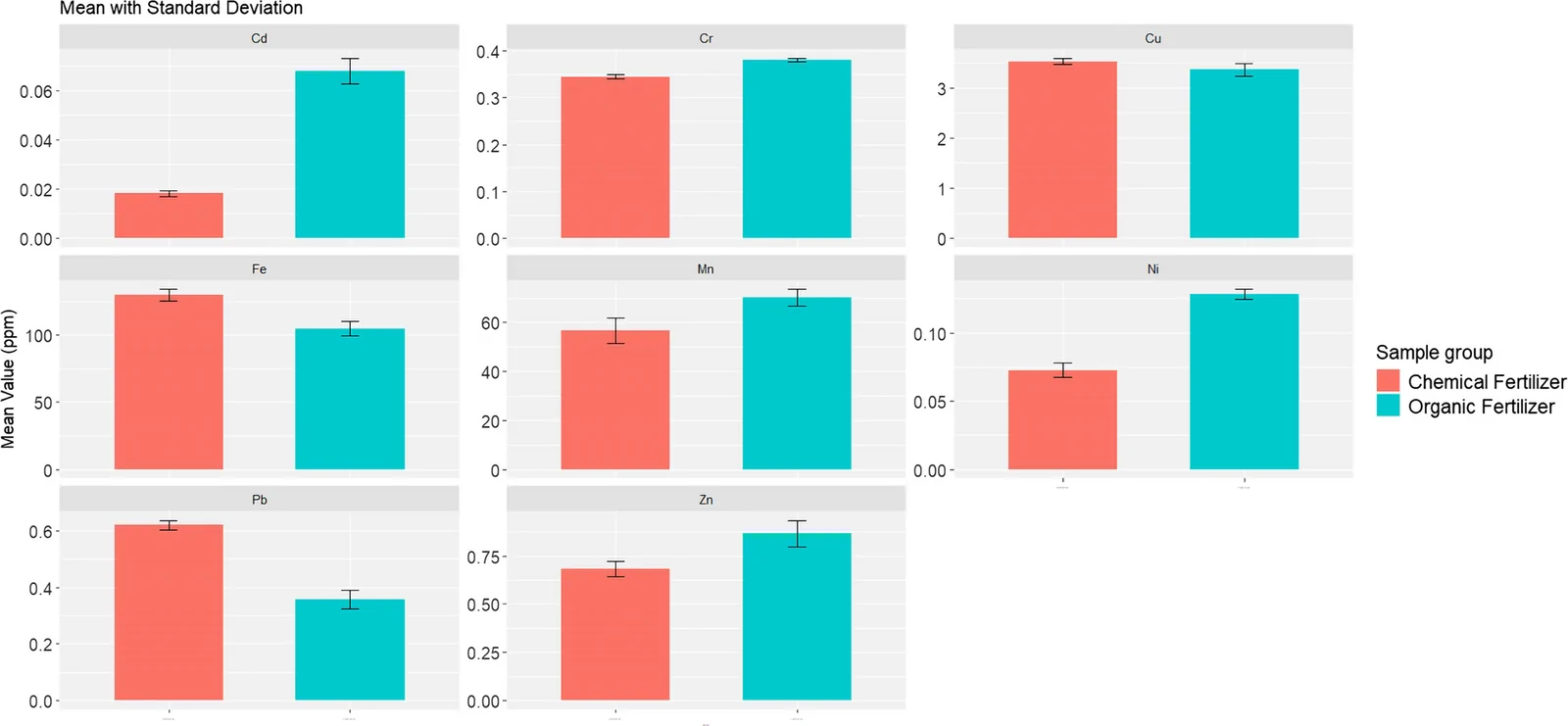
The heavy metal analysis of the soil samples including chemical and organic fertilizer groups. *p* value < 0.05 implies a statistically significant difference between the groups, where the number of asterisks (∗*p* < 0.05, ∗∗*p* < 0.01, and ∗∗∗*p* < 0.001) confers the magnitude of the differences

Statistically significant differences in heavy metal concentrations were identified between chemical and organic fertilizer treatments (*p* < 0.001 for all parameters). Pb and Fe concentrations were higher in soils treated with chemical fertilizers (0.62 ± 0.02 and 129.83 ± 4.26, respectively) compared to those treated with organic fertilizer (0.36 ± 0.03 and 104.83 ± 5.19, respectively). Conversely, Cd, Ni, Cr, Mn, and Zn concentrations were greater in organically fertilized soils (0.068 ± 0.01, 0.13 ± 0.004, 0.38 ± 0.003, 70.17 ± 3.37, and 0.87 ± 0.07, respectively) than in soils receiving chemical fertilizers. Cu concentrations were comparable between the two fertilizer regimes, with values of 3.52 ± 0.06 for chemical and 3.36 ± 0.12 for organic fertilizer (Fig. 1, Data S1).

Significant differences in soil physicochemical properties were observed between the fertilizer treatments. Application of organic fertilizer resulted in higher organic matter (1.98 ± 0.14) and organic carbon (1.13 ± 0.08) levels compared to chemical fertilizer (1.49 ± 0.07 and 0.86 ± 0.05, respectively; *p* < 0.001). Soil pH was also elevated under organic fertilizer (5.90 ± 0.09) relative to chemical fertilizer (5.57 ± 0.08; *p* < 0.001). In contrast, EC did not differ significantly between the two treatments (Table 2, Data S1).

**Table 2:** The physicochemical properties of soil samples including chemical and organic fertilizer groups

Physicochemical properties	Soil treatment type		*p* value
	Chemical fertilizer (%mean ± SD)	Organic fertilizer (%mean ± SD)	
OM (%)	1.49 ± 0.07	1.98 ± 0.14	<0.001
OC (%)	0.86 ± 0.05	1.13 ± 0.08	<0.001
pH	5.57 ± 0.08	5.90 ± 0.09	<0.001
EC (dS/m)	0	0	–

Note: The *p* value < 0.05 implies a statistically significant difference

### Bacteriome diversity and composition of soil samples

Twelve soil samples were analyzed and categorized into two groups: chemical fertilizer (CF1–6) and organic fertilizer (OF1–6). Oxford Nanopore long-read 16S rRNA gene sequencing was employed to elucidate bacteriome signatures and diversity. Table S1 provides detailed information on the sampling methodology, including sample descriptions, sources and locations, amplicon sequence data, taxonomic features assigned to each sample, and the corresponding sequence read archive (SRA) accession numbers.

**Fig. 2:**
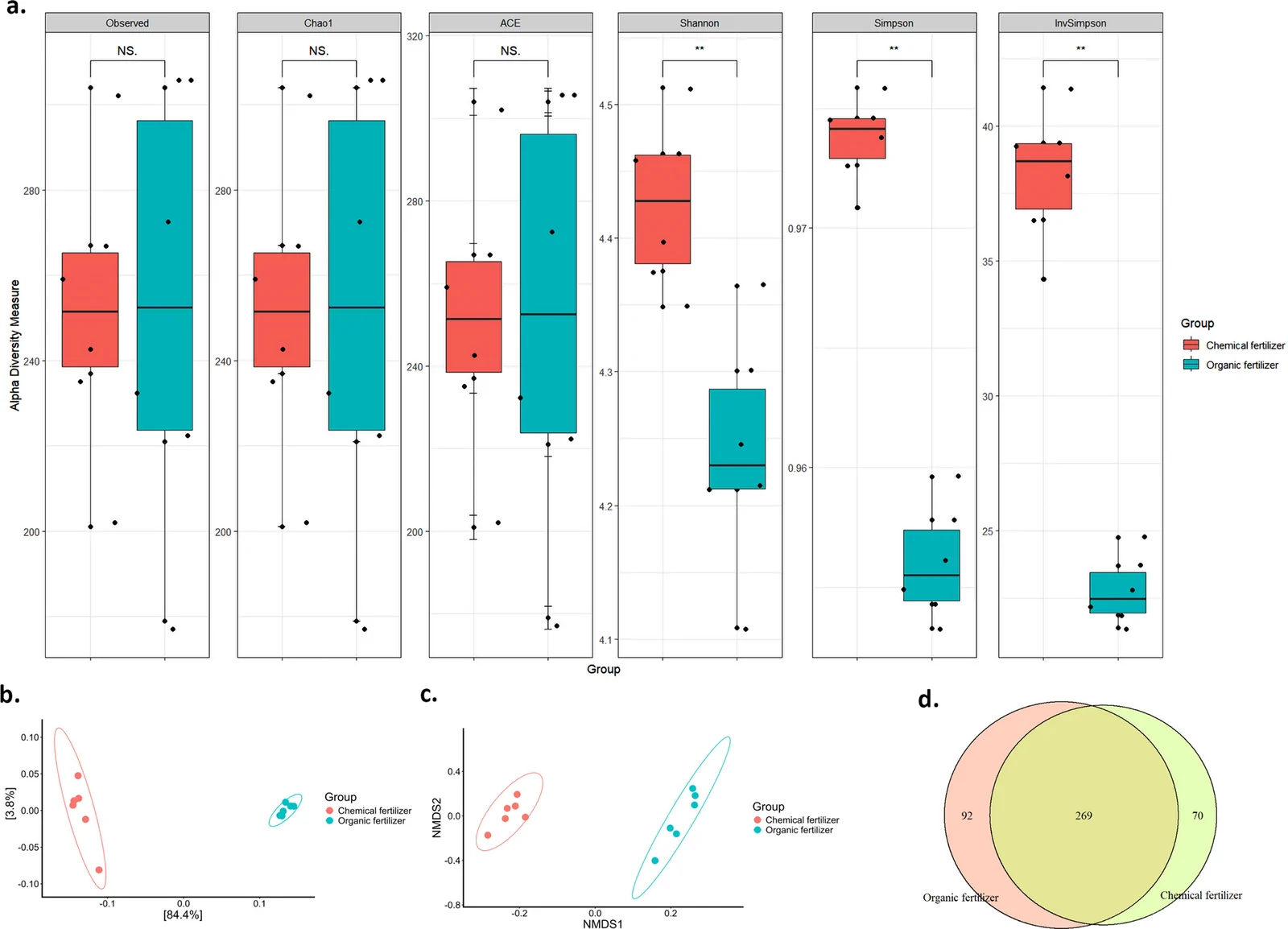
Bacteriome diversity and composition of Soil samples. **a** Alpha diversity measure within the sample groups (chemical fertilizer: CF1–6; organic fertilizer: OF1–6), using the observed, Chao1, ACE, Shannon, Simpson, and InvSimpson measures. The data are presented in the form of boxplots, and Wilcoxon rank-sum tests (∗*p* < 0.05) are used to compare them. **b** and **c** Beta diversity among the sample groups was assessed using the Bray–Curtis dissimilarity distance method. Results were visualized through Principal Coordinate Analysis (PCoA) and non-metric multidimensional scaling (NMDS) plots. Samples were color-coded by category and matched to the corresponding ellipses. A PERMANOVA test, employing a simplified model and pairwise comparisons on the distance matrix, indicated that bacterial communities differed significantly among the sample groups (*p *value = 0.003, *R*^2^ = 0.84016). **d** The observed number of taxonomic features included 431 bacterial genera

To assess whether soil bacteriome diversity differed between chemical fertilizer (CF1–6) and organic fertilizer (OF1–6) treatments, both within-sample (alpha) and between-sample (beta) diversities of the bacterial communities were analyzed. The observed species, Chao1, and ACE indices indicated no significant differences (*p *value > 0.05 Wilcoxon rank-sum test). In contrast, the Shannon, Simpson, and InvSimpson indices revealed significant differences in bacterial community richness and evenness (*p *value < 0.05, Wilcoxon rank-sum test) (Fig. 2a). Principal Coordinate Analysis (PCoA) and non-metric multidimensional scaling (NMDS) based on Bray–Curtis dissimilarity distance indicated a significant difference in bacteriome composition among sample groups (*p *value = 0.003, *R*^2^ = 0.84016, PERMANOVA test) (Fig. 2b and c). A total of 431 taxonomic features were identified across the sample groups. Of these, 269 features were shared, while 92 were unique to the organic fertilizer group and 70 were unique to the chemical fertilizer group. The analysis also identified 19 bacterial phyla and 431 genera (Fig. 2d).

### Relative abundances of bacteriomes in soil samples

**Fig. 3:**
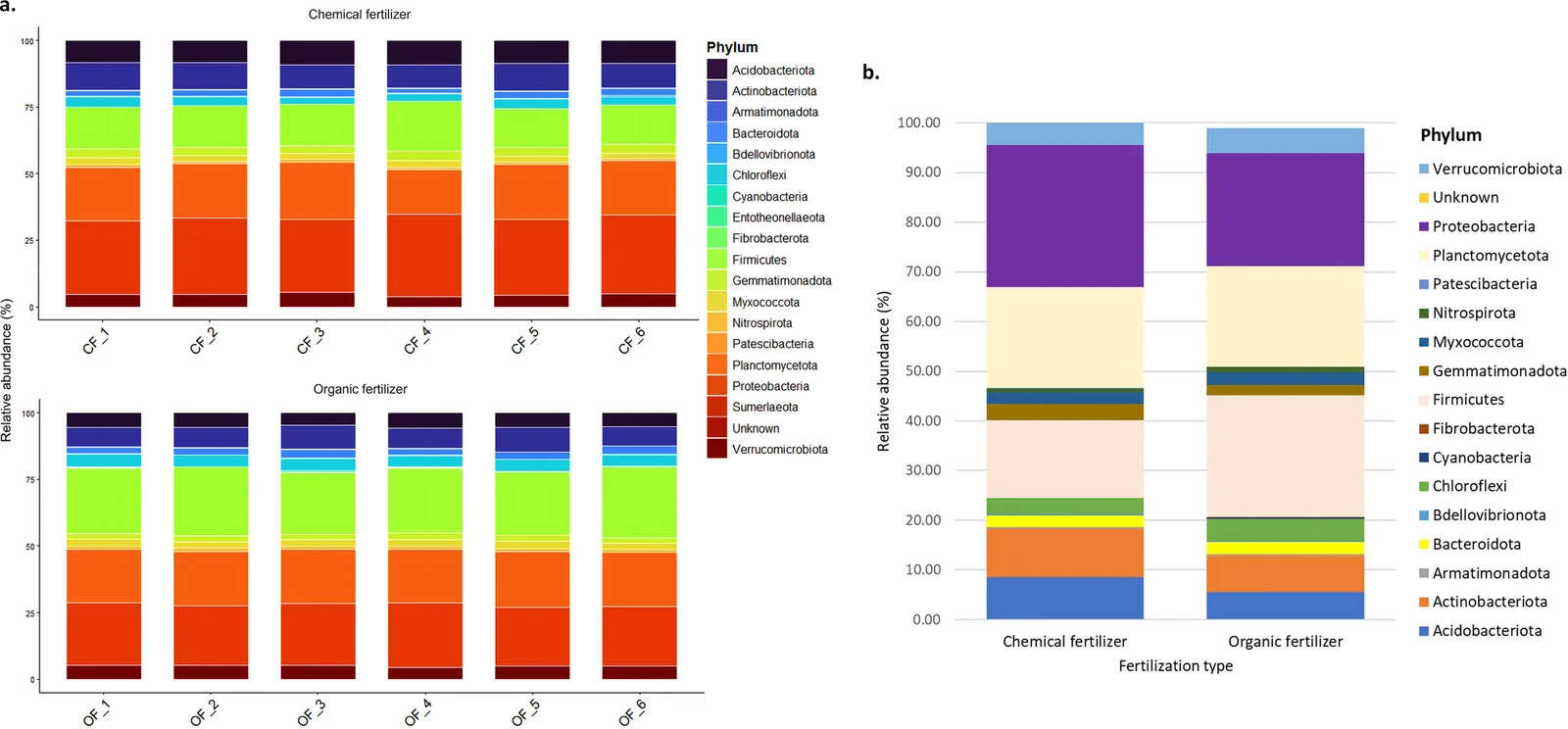
The relative abundance of bacteriome in soil samples at phylum level. Stacked bar plots showing the relative abundance of bacterial phyla in **a** individual samples and **b** sample groups across the respective categories

At the phylum level, soils treated with organic fertilizer were primarily dominated by Firmicutes (24.57%), Proteobacteria (22.76%), and Planctomycetota (20.29%). Moderate abundances were observed for Actinobacteriota (7.48%), Acidobacteriota (5.49%), Verrucomicrobiota (5.02%), and Chloroflexi (4.36%). Lower relative abundances were detected for Myxococcota (2.55%), Bacteroidota (2.51%), and Gemmatimonadota (2.04%). In soils treated with chemical fertilizers, Proteobacteria were the most abundant (28.58%), followed by Planctomycetota (20.29%) and Firmicutes (15.63%). Actinobacteriota (9.72%) and Acidobacteriota (8.58%) exhibited moderate relative abundances. Verrucomicrobiota (4.54%), Chloroflexi (3.20%), Gemmatimonadota (3.19%), Bacteroidota (2.37%), and Myxococcota (2.35%) were present in lower abundances. (Fig. 3, Data S2).

**Fig. 4:**
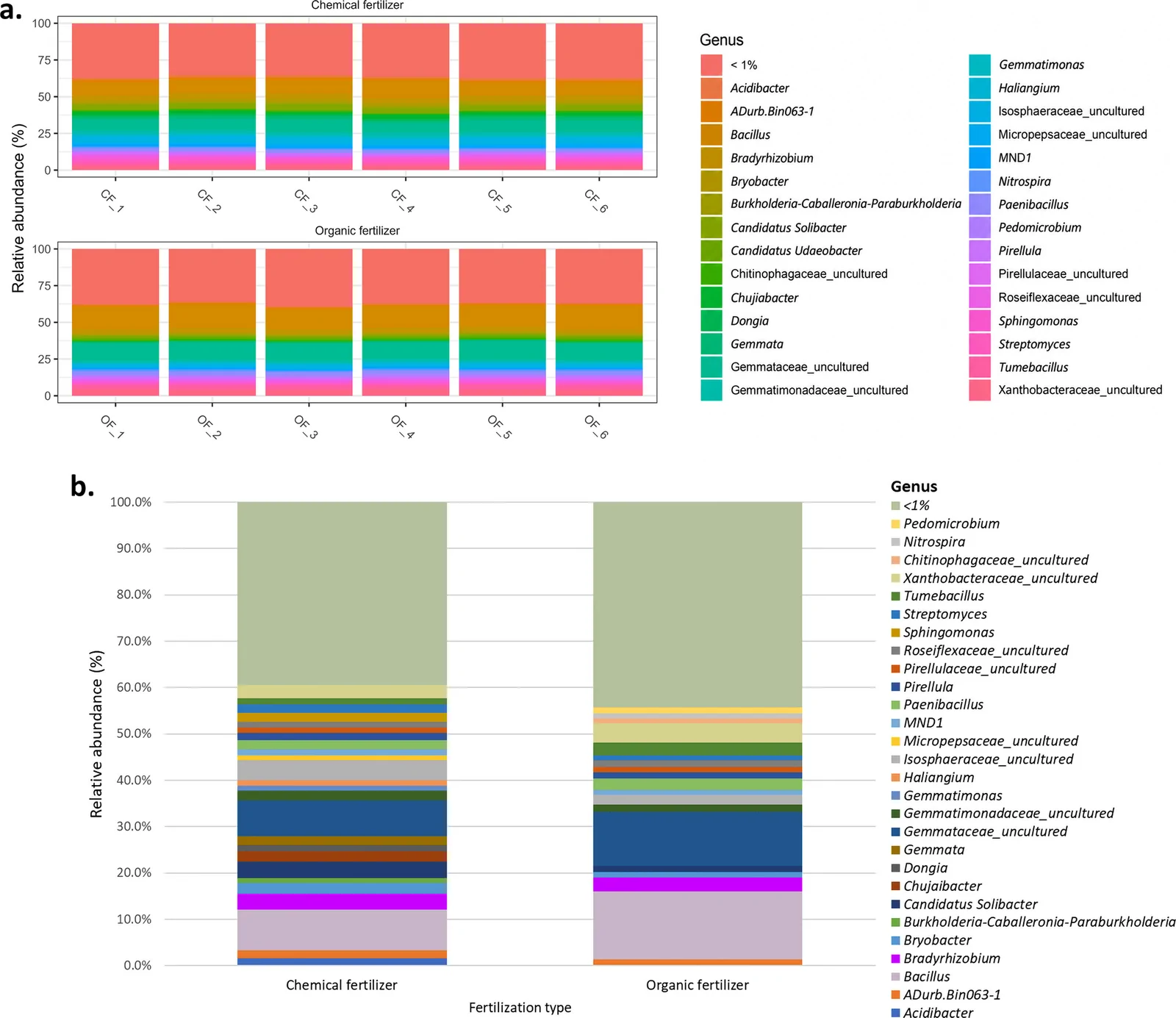
The relative abundance of bacteria taxa at genus level. Stacked bar plots showing the relative abundance of bacterial genera in **a** individual samples and **b** sample groups across the respective categories

In organic fertilized soil samples, *Bacillus* (14.77%) was the most abundant genus, followed by Gemmataceae_uncultured (11.60%). Lower relative abundances were recorded for Xanthobacteraceae_uncultured (4.15%), *Bradyrhizobium* (2.90%), *Tumebacillus* (2.73%), *Paenibacillus* (2.50%), and Isosphaeraceae_uncultured (2.14%). In chemically fertilized soils, *Bacillus* (8.84%) was the most abundant genus, followed by Gemmataceae_uncultured (7.65%) and Isosphaeraceae_uncultured (4.30%). Additional genera detected included *Candidatus* Solibacter (3.53%), *Bradyrhizobium* (3.33%), Xanthobacteraceae_uncultured (2.88%), *Bryobacter* (2.38%), *Chujaibacter* (2.21%), Gemmatimonadaceae_uncultured (2.12%), *Paenibacillus* (2.02%), and *Gemmata* (2.01%) (Fig. 4, Data S2). Among the detected taxa, three genera, including *Bacillus*, *Bradyrhizobium*, and *Paenibacillus*—were identified as the principal PGPB taxa based on their well-established roles in plant growth promotion. For Gemmataceae_uncultured, Xanthobacteraceae_uncultured, Isosphaeraceae_uncultured, and Gemmatimonadaceae_uncultured, the database did not provide reliable genus level assignments. Therefore, these taxa were conservatively retained at the family level and were not assigned to a genus beyond the resolution supported by the database.

A comparison of the shared and most abundant bacterial genera among the sample groups identified significant differences. Several bacterial taxa demonstrated significantly higher relative abundance in soils subjected to organic fertilization compared to those managed with chemical fertilizers. The genus *Bacillus* showed substantially higher abundance under organic fertilization (14.77 ± 0.790%) than under chemical fertilization (8.84 ± 1.255%; FDR < 0.001). Similarly, Gemmataceae_uncultured was more abundant in organically fertilized soils (11.60 ± 0.441%) than in chemically fertilized soils (7.65 ± 0.689%; FDR < 0.001), while Xanthobacteraceae_uncultured exhibited a comparable pattern, with abundances of 4.15 ± 0.204% and 2.88 ± 0.197%, respectively (FDR < 0.001). The genera *Tumebacillus* and *Paenibacillus* were also significantly more abundant under organic fertilization, with mean abundances of 2.73 ± 0.183% versus 1.35 ± 0.171% and 2.50 ± 0.155% versus 2.02 ± 0.173%, respectively (FDR < 0.001).

In contrast, several taxa exhibited higher abundance under chemical fertilization. *Bradyrhizobium* showed a lower but significant increase under chemical fertilization (3.33 ± 0.240%) compared with organic fertilization (2.90 ± 0.216%; FDR = 0.055). Similarly, Isosphaeraceae_uncultured was more abundant in chemically fertilized soils (4.30 ± 0.365%) than in organically fertilized soils (2.14 ± 0.224%; FDR < 0.001), while Gemmatimonadaceae_uncultured showed a smaller but significant difference, with abundances of 2.12 ± 0.311% and 1.56 ± 0.160% under chemical and organic fertilization, respectively (FDR = 0.004). *Pirellula* was also slightly more abundant under chemical fertilization (1.54 ± 0.150%) than organic fertilization (1.28 ± 0.125%; FDR = 0.076). In contrast, the difference in Roseiflexaceae_uncultured abundance between chemical (1.26 ± 0.340%) and organic (1.41 ± 0.156%) fertilization was not statistically significant (FDR = 0.368) (Table 3).

**Table 3:** The predominant bacterial genus found in the sample groups

Bacterial taxa	Relative abundance of bacteria (%)		FDR (*q* value)
	Chemical fertilizer (mean ± SD)	Organic fertilizer (mean ± SD)	
*Bacillus*	8.84 ± 1.255	14.77 ± 0.790	<0.001
Gemmataceae_uncultured	7.65 ± 0.689	11.60 ± 0.441	<0.001
Xanthobacteraceae_uncultured	2.88 ± 0.197	4.15 ± 0.204	<0.001
*Bradyrhizobium*	3.33 ± 0.240	2.90 ± 0.216	0.055
*Tumebacillus*	1.35 ± 0.171	2.73 ± 0.183	<0.001
*Paenibacillus*	2.02 ± 0.173	2.50 ± 0.155	<0.001
Isosphaeraceae_uncultured	4.30 ± 0.365	2.14 ± 0.224	<0.001
Gemmatimonadaceae_uncultured	2.12 ± 0.311	1.56 ± 0.160	0.004
Roseiflexaceae_uncultured	1.26 ± 0.340	1.41 ± 0.156	0.368
*Pirellula*	1.54 ± 0.150	1.28 ± 0.125	0.076

Note: The FDR (*q* value) < 0.05 implies a statistically significant difference

### Functional profile of bacterial communities in chemical and organic fertilizers

**Fig. 5:**
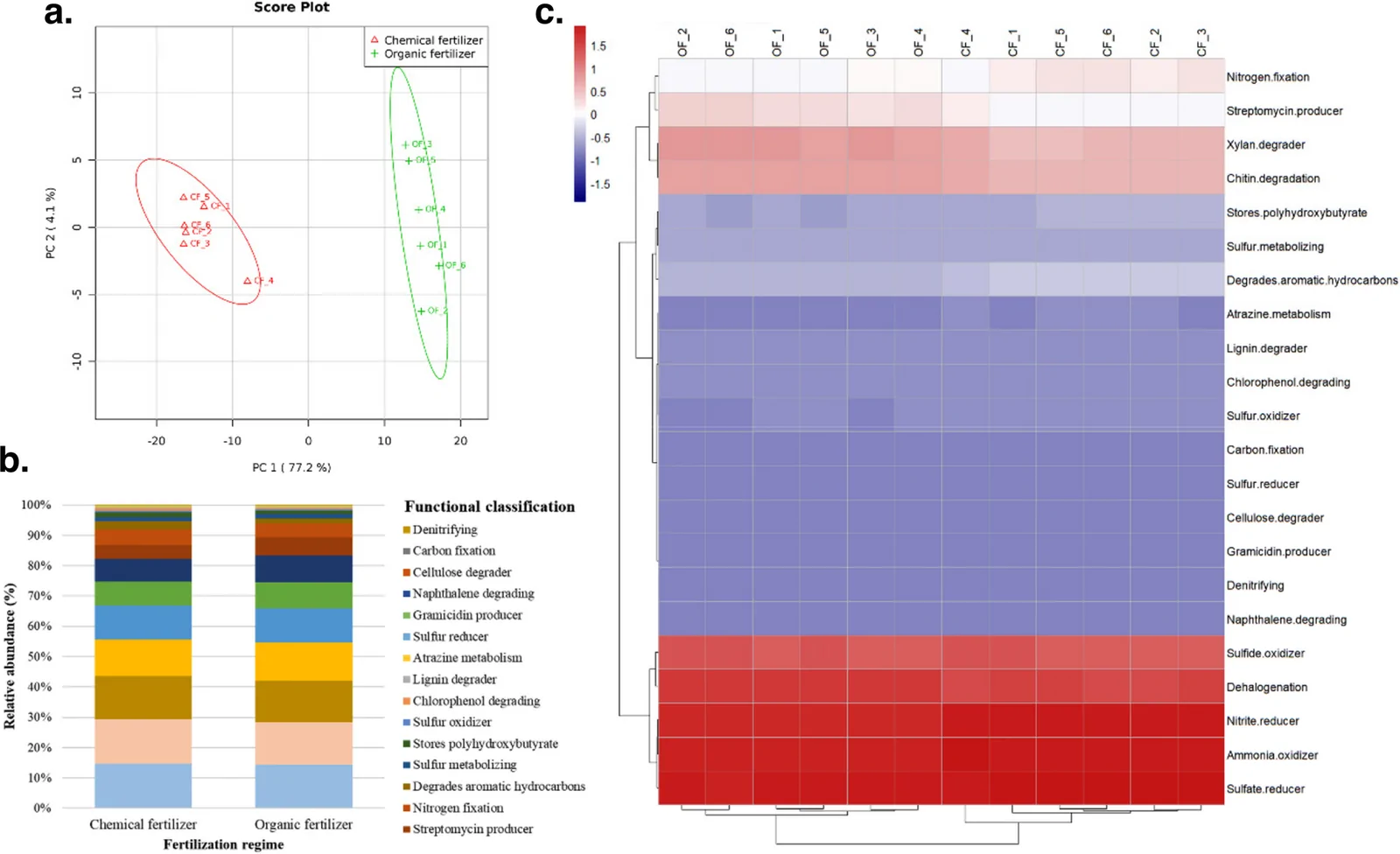
Functional-based classification of bacterial communities in chemical and organic fertilizers. **a** Principal Component Analysis (PCA) score plot showing the distribution of bacteriome functional profiles across individual samples in the respective treatment groups. **b** Stacked bar plots illustrating the relative abundance of bacterial genera at the group level. **c** Heatmap displays the relative abundances and hierarchical grouping of the functional profiles (*n* = 22) in the individual samples. Red cells indicate more prevalent patterns, while blue cells denote less abundant taxa within each sample

Functional profiling of the bacterial communities showed distinct clusters in PCA, separating samples by chemical and organic fertilizer treatments (Fig. 5a). In this analysis, the bacterial community under chemical fertilization showed relatively higher representation of functional groups associated with nutrient cycling and redox processes. Sulfate-reducing bacteria accounted for the highest relative proportion (14.77%), followed by ammonia oxidizers (14.51%) and nitrite reducers (14.33%) (Fig. 5b, Data S3). Dehalogenation-related functions accounted for 12.04% of the community, while sulfide-oxidizing functions represented 11.24%. Carbohydrate-degrading functions, including chitin degradation (7.78%) and xylan degradation (7.62%), were also detected. Streptomycin producers (4.51%) and Nitrogen-fixing bacteria (5.25%) occurred at comparatively lower proportions. Functions related to aromatic hydrocarbon degradation (2.59%), sulfur metabolism (1.33%), and polyhydroxybutyrate storage (1.60%) were detected at relatively low proportions (Fig. 5b, Data S3).

The bacterial community under organic fertilization showed relatively higher representation of functions associated with nutrient transformation and organic matter degradation. Sulfate-reducing bacteria represented the largest proportion (14.33%), followed by ammonia oxidizers (14.02%) and nitrite reducers (13.67%). Dehalogenation-related functions accounted for 12.64%, while sulfide-oxidizing bacteria represented 11.28%. Functions associated with organic matter degradation, including xylan degradation (8.86%) and chitin degradation (8.58%), were also detected at comparatively higher proportions (Fig. 5b, Data S3). Streptomycin-producing bacteria accounted for 6.09% of the community. However, nitrogen-fixing microorganisms were predicted at a lower abundance (4.47%). In contrast, functions related to aromatic hydrocarbon degradation (1.73%), sulfur metabolizing pathways (1.44%), and polyhydroxybutyrate storage (1.17%) were relatively infrequent (Fig. 5b, Data S3).

### Correlation between predominant taxa and soil properties

**Fig. 6:**
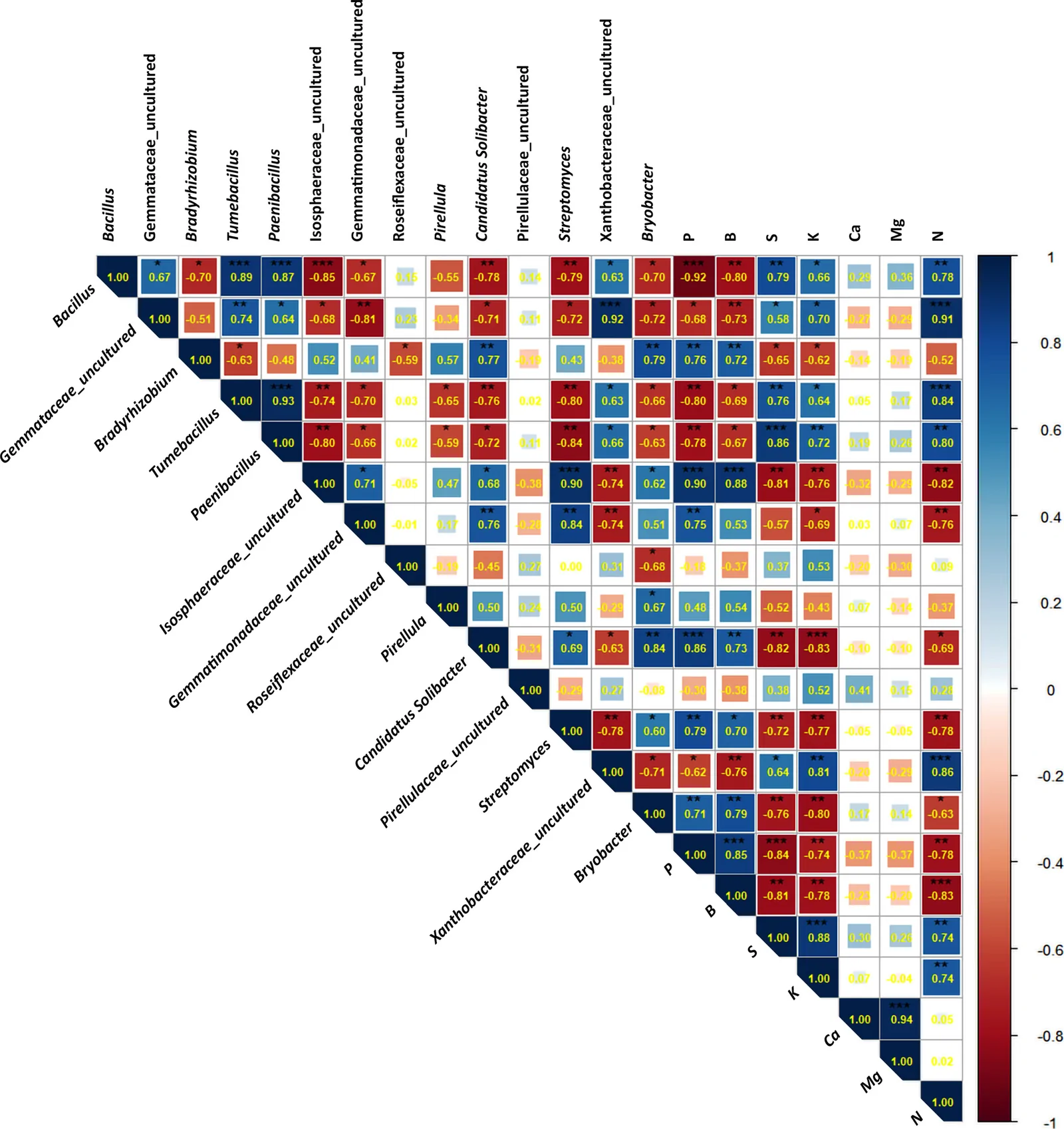
The correlation coefficient between predominant taxa and soil nutritional properties. The correlation matrix illustrates relationships ranging from −1 to +1, where blue-colored cells indicate strong correlations, whereas red-colored cells represent weak correlations. *p* value < 0.05 implies a statistically significant difference between the groups, where the number of asterisks (∗*p* < 0.05, ∗∗*p* < 0.01, and ∗∗∗*p* < 0.001) confers the magnitude of the differences

Spearman’s rank correlation coefficient analysis revealed that several soil physicochemical properties were strongly and significantly associated with specific bacterial taxa. Among the measured nutrients, P exhibited particularly strong positive correlations with multiple bacterial groups, including Isosphaeraceae_uncultured (*r*_*s*_ = 0.90, *p* < 0.001), *Bradyrhizobium* (*r*_*s*_ = 0.76, *p* < 0.01), Gemmatimonadaceae_uncultured (*r*_*s*_ = 0.75, *p* < 0.01), *Candidatus Solibacter* (*r*_*s*_ = 0.86, *p* < 0.001), *Streptomyces* (*r*_*s*_ = 0.79, *p* < 0.01), and *Bryobacter* (*r*_*s*_ = 0.71, *p* < 0.01). Similarly, B exhibited strong positive associations with several of the same bacterial groups. Significant correlations were identified with *Bradyrhizobium* (*r*_*s*_ = 0.72, *p* < 0.01), Isosphaeraceae_uncultured (*r*_*s*_ = 0.88, *p* < 0.001), *Candidatus* Solibacter (*r*_*s*_ = 0.73, *p* < 0.01), *Streptomyces* (*r*_*s*_ = 0.70, *p* < 0.05), and *Bryobacter* (*r*_*s*_ = 0.79, *p* < 0.01). In addition to phosphorus and boron, S showed strong positive correlations with several well-characterized soil bacterial genera, including *Bacillus* (*r*_*s*_ = 0.79, *p* < 0.01), *Tumebacillus* (*r*_*s*_ = 0.76, *p* < 0.01), and *Paenibacillus* (*r*_*s*_ = 0.86, *p* < 0.001). K also displayed strong positive relationships with Gemmataceae_uncultured (*r*_*s*_ = 0.70, *p* < 0.05), *Paenibacillus* (*r*_*s*_ = 0.72, *p* < 0.01), and Xanthobacteraceae_uncultured (*r*_*s*_ = 0.81, *p* < 0.001). Additionally, N was strongly correlated with several bacterial taxa, including *Bacillus* (*r*_*s*_ = 0.78, *p* < 0.01), Gemmataceae_uncultured (*r*_*s*_ = 0.91, *p* < 0.001), *Tumebacillus* (*r*_*s*_ = 0.84, *p* < 0.001), *Paenibacillus* (*r*_*s*_ = 0.80, *p* < 0.01), and Xanthobacteraceae_uncultured (*r*_*s*_ = 0.86, *p* < 0.001). In contrast, Ca and Mg exhibited only weak positive correlations (*r*_*s*_ < 0.3) with most bacterial taxa (Fig. 6).

**Fig. 7:**
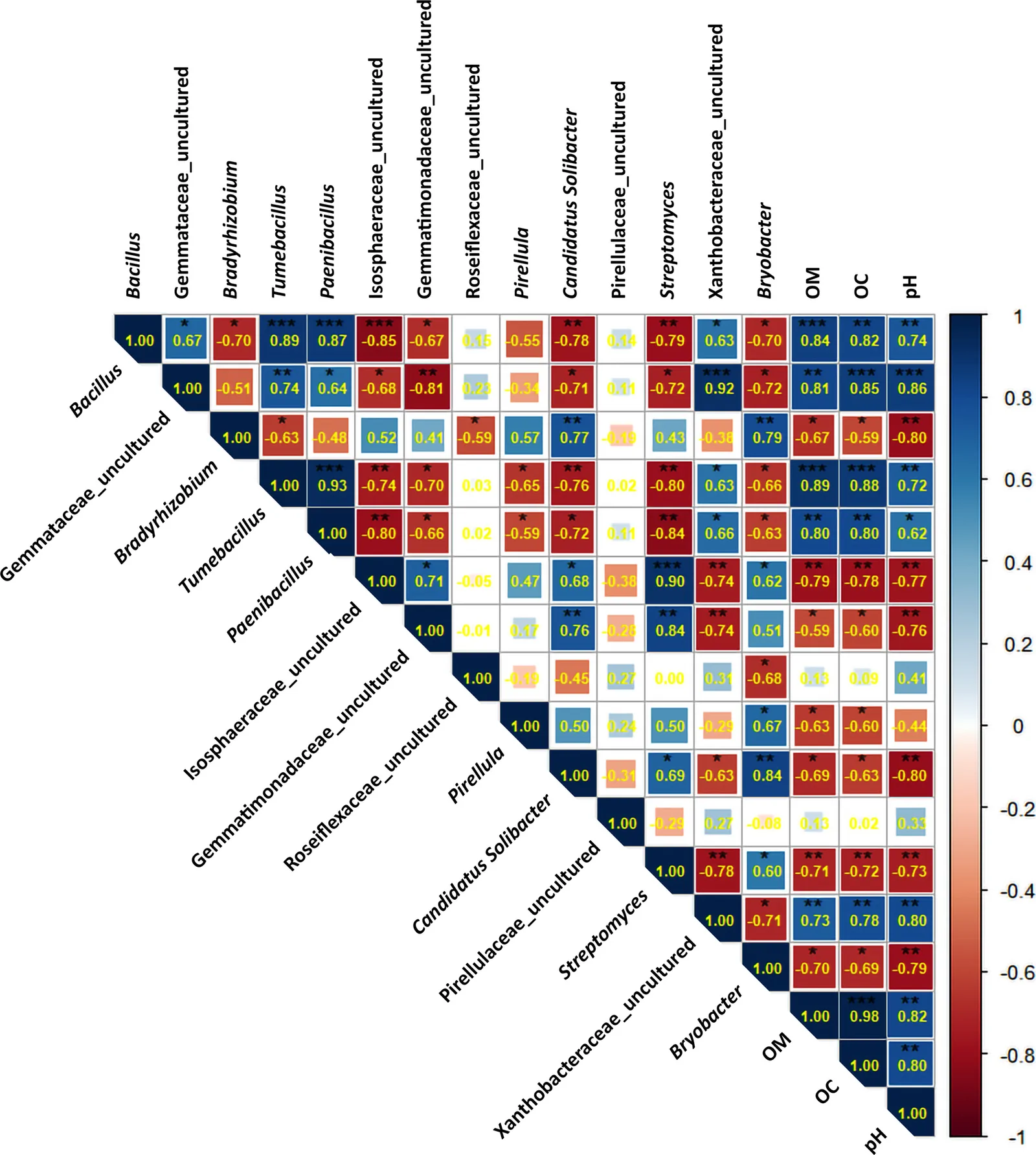
The correlation coefficient between predominant taxa and soil physicochemical properties. The correlation matrix illustrates relationships ranging from −1 to +1, where blue-colored cells indicate strong correlations, whereas red-colored cells represent weak correlations. *p* value < 0.05 implies a statistically significant difference between the groups, where the number of asterisks (∗*p* < 0.05, ∗∗*p* < 0.01, and ∗∗∗*p* < 0.001) confers the magnitude of the differences

Spearman’s rank correlation coefficient analysis revealed significant associations between Soil OM and several dominant bacterial taxa. OM showed strong positive correlations with *Bacillus* (*r*_*s*_ = 0.84, *p* < 0.001), Gemmataceae_uncultured (*r*_*s*_ = 0.81, *p* < 0.001), *Tumebacillus* (*r*_*s*_ = 0.89, *p* < 0.001), *Paenibacillus* (*r*_*s*_ = 0.80, *p* < 0.01), and Xanthobacteraceae_uncultured (*r*_*s*_ = 0.73, *p* < 0.01). A comparable pattern was identified for OC, which also exhibited strong positive correlations with several of the same bacterial taxa. Significant associations were found with *Bacillus* (*r*_*s*_ = 0.82, *p* < 0.01), Gemmataceae_uncultured (*r*_*s*_ = 0.85, *p* < 0.001), *Tumebacillus* (*r*_*s*_ = 0.88, *p* < 0.001), *Paenibacillus* (*r*_*s*_ = 0.80, *p* < 0.01), and Xanthobacteraceae_uncultured (*r*_*s*_ = 0.78, *p* < 0.01). In addition to organic parameters, Soil pH also exceeded organic parameters; it demonstrated significant positive relationships with several bacterial taxa. Strong correlations were observed with *Bacillus* (*r*_*s*_ = 0.82, *p* < 0.01), Gemmataceae_uncultured (*r*_*s*_ = 0.86, *p* < 0.001), *Tumebacillus* (*r*_*s*_ = 0.72, *p* < 0.01), and Xanthobacteraceae_uncultured (*r*_*s*_ = 0.80, *p* < 0.01) (Fig. 7).

**Fig. 8:**
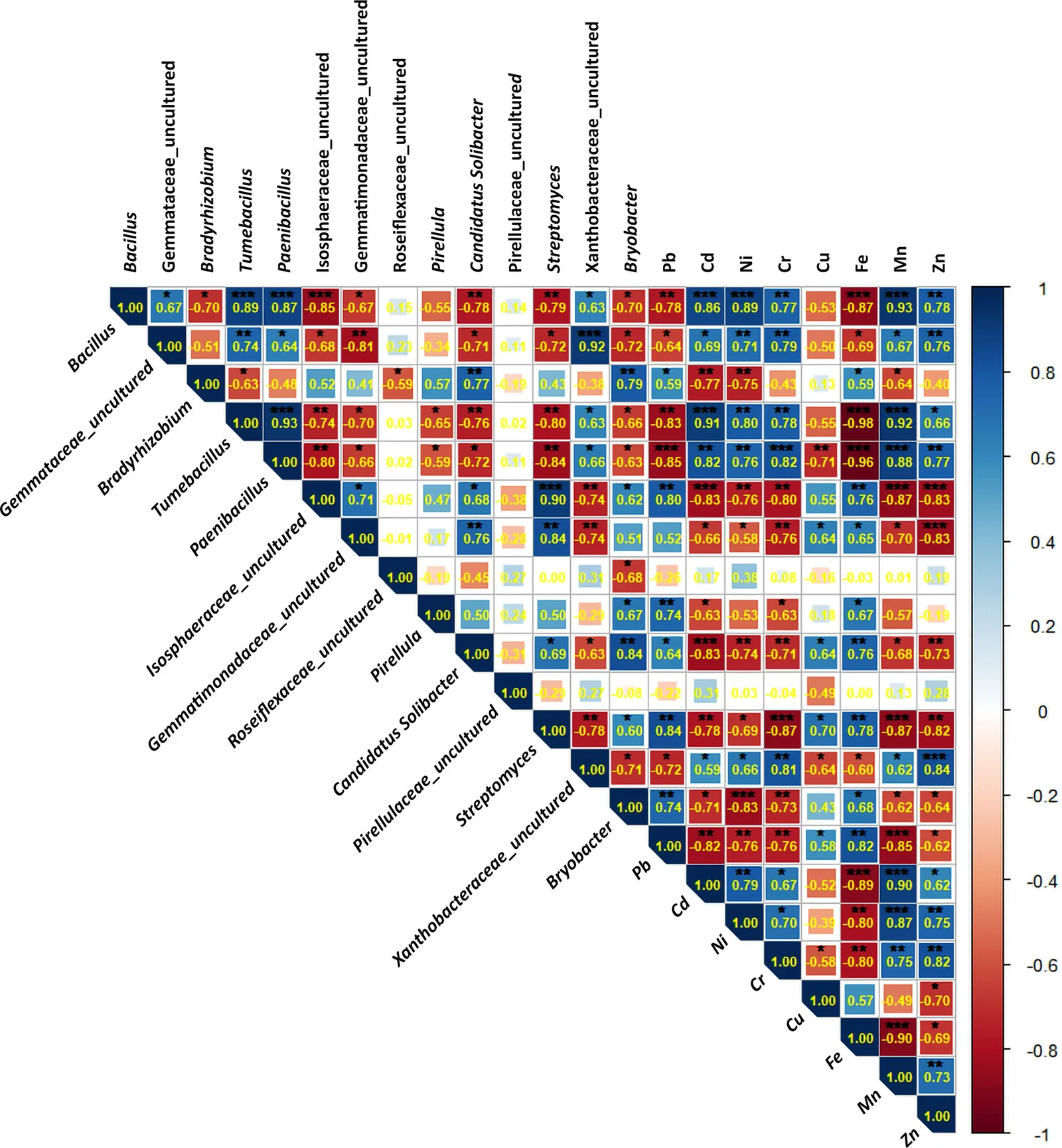
The correlation coefficient between predominant taxa and soil heavy metals. The correlation matrix illustrates relationships ranging from −1 to +1, where blue-colored cells indicate strong correlations, whereas red-colored cells represent weak correlations. *p* value < 0.05 implies a statistically significant difference between the groups, where the number of asterisks (∗*p* < 0.05, ∗∗*p* < 0.01, and ∗∗∗*p* < 0.001) confers the magnitude of the differences

The analysis identified significant associations between Soil trace elements and dominant bacterial taxa. Among the heavy metal examined, Pb exhibited strong positive correlations with several bacterial groups, including Isosphaeraceae_uncultured (*r*_*s*_ = 0.80, *p* < 0.01), *Pirellula* (*r*_*s*_ = 0.74, *p* < 0.01), *Streptomyces* (*r*_*s*_ = 0.84, *p* < 0.01), and *Bryobacter* (*r*_*s*_ = 0.74, *p* < 0.01). Cd also showed strong positive associations with several bacterial genera, particularly *Bacillus* (*r*_*s*_ = 0.86, *p* < 0.001), *Tumebacillus* (*r*_*s*_ = 0.91, *p* < 0.001), and *Paenibacillus* (*r*_*s*_ = 0.82, *p* < 0.01) (Fig. 8). Similarly, Ni demonstrated strong positive correlations with *Bacillus* (*r*_*s*_ = 0.89, *p* < 0.001), Gemmataceae_uncultured (*r*_*s*_ = 0.71, *p* < 0.01), *Tumebacillus* (*r*_*s*_ = 0.80, *p* < 0.01), and *Paenibacillus* (*r*_*s*_ = 0.76, *p* < 0.01). Cr also exhibited strong positive relationships with several bacterial groups, including *Bacillus* (*r*_*s*_ = 0.77, *p* < 0.01), Gemmataceae_uncultured (*r*_*s*_ = 0.79, *p* < 0.01), *Tumebacillus* (*r*_*s*_ = 0.78, *p* < 0.01), *Paenibacillus* (*r*_*s*_ = 0.82, *p* < 0.001), and Xanthobacteraceae_uncultured (*r*_*s*_ = 0.81, *p* < 0.01) (Fig. 8). In contrast, Cu showed fewer positive associations with the bacterial taxa analyzed. In addition, Fe demonstrated several significant positive correlations, particularly with Isosphaeraceae_uncultured (*r*_*s*_ = 0.76, *p* < 0.01), *Candidatus* Solibacter (*r*_*s*_ = 0.76, *p* < 0.01), and *Streptomyces* (*r*_*s*_ = 0.78, *p* < 0.01). Similarly, Mn exhibited very strong positive relationships with *Bacillus* (*r*_*s*_ = 0.93, *p* < 0.001), *Tumebacillus* (*r*_*s*_ = 0.92, *p* < 0.001), and *Paenibacillus* (*r*_*s*_ = 0.88, *p* < 0.001). Zn also demonstrated strong positive correlations with several bacterial groups, including *Bacillus* (*r*_*s*_ = 0.78, *p* < 0.01), Gemmataceae_uncultured (*r*_*s*_ = 0.76, *p* < 0.01), *Paenibacillus* (*r*_*s*_ = 0.77, *p* < 0.01), and Xanthobacteraceae_uncultured (*r*_*s*_ = 0.84, *p* < 0.001) (Fig. 8).

## Discussion

A comprehensive understanding of soil bacterial community composition is essential for evaluating soil health, nutrient-use efficiency, and the long-term sustainability of agricultural systems. Soil functions are mediated by complex and predominantly uncultivable microbial assemblages [1]. Prolong application of chemical fertilization is frequently used to increase agricultural productivity by providing vital nutrients. Several studies have found that excessive nitrogen fertilization lowers microbial diversity and changes bacterial and fungal community structures toward taxa suited to nutrient-rich circumstances, while suppressing beneficial microbes engaged in nutrient cycle [80, 81]. Although chemical fertilizers improve nutrient availability, their prolong usage often reduces soil organic carbon (SOC), which is the principal energy source for soil microbes. Declining SOC inhibits microbial growth and biomass, particularly when agricultural leftovers are removed from fields. As a result, microbial-mediated activities including carbon sequestration, mineralization, and aggregate formation are less efficient [80]. Soil enzymatic activity is also impacted by prolong application of fertilization. When compared to integrated nutrient management systems that blend mineral fertilizers with organic amendments, continuous inorganic fertilization often lowers enzyme activity [82].

High-throughput 16S rRNA gene sequencing offers a reliable, culture-independent method for characterizing soil bacterial communities, providing precise taxonomic resolution, reliable diversity assessments, and the ability to detect shifts in microbial structure resulting from fertilization practices [43]. The present study evaluates the soil bacterial community under long-term chemical and organic fertilization regimes using long-read 16S rRNA gene sequencing based on Oxford Nanopore technology. The study reveals that prolong fertilization regime influences soil bacterial community structure and diversity. SILVA database was employed to characterize the bacteriome of soils treated with chemical and organic fertilizers, resulting in the identification of 431 genera. While alpha diversity metrics did not differ significantly between treatments, indices accounting for both richness and evenness (Shannon, Simpson, InvSimpson) indicated a significant (*p* < 0.05) increase in bacterial diversity under chemical fertilization [83]. These indices are widely applied in microbial ecology as they offer a comprehensive representation of community structure by considering both the number of taxa present and their relative abundance and distribution. The Shannon index is sensitive to both rare and abundant taxa, while Simpson-based indices emphasize patterns of community dominance and evenness [73, 74]. Therefore, increases in these indices indicate a more balanced and evenly distributed microbial community under chemical fertilization, even when richness-based metrics remain unchanged. These diversity metrics serve as important indicators of microbial community stability and ecosystem functioning in soil environments [84]. Beta diversity analyses confirmed distinct community compositions between the two fertilizer regimes, with organic amendments supporting a unique assemblage of 92 taxonomic features compared to 70 features unique to chemical fertilizer soils. These findings imply that the structure of bacterial communities may be differently shaped by chemical and organic fertilization, yet the observed alpha diversity patterns do not show increased diversity under organic fertilization. Such differences in community composition may influence soil microbial functions and, consequently, contribute to soil fertility and sustainability over time [85].

In this study, soil treated with organic fertilization showed increased abundances of *Bacillus*, Gemmataceae, Xanthobacteraceae, *Tumebacillus*, and *Paenibacillus* compared to chemically fertilized soil. Many of these taxa are recognized for promoting soil fertility and plant growth. *Bacillus*, *Paenibacillus*, *Bradyrhizobium*, and *Tumebacillus*, for example, are well-known PGPB that support plant productivity through nutrient solubilization, phytohormone production, and systemic resistance in host plants [86, 87]. *Bacillus* promotes plant growth through phosphate solubilization, phytohormone production, siderophore-mediated nutrient acquisition, biocontrol, and stress tolerance [14, 15]. *Bradyrhizobium* contributes primarily through symbiotic nitrogen fixation and stress mitigation [16]. On the other hand, *Paenibacillus* enhances plant growth via nitrogen fixation, phosphate solubilization, indole-3-acetic acid and siderophore production, and pathogen suppression [17]. The higher abundance of these genera in organically fertilized soils indicates that prolong application of organic amendments may foster beneficial rhizosphere microorganisms that enhance plant development and nutrient availability [88].

The higher abundance of Xanthobacteraceae in organically managed soils likely contributes to enhanced soil functionality. Members of this family perform nitrogen-fixation and degrade aromatic compounds, thereby facilitating nutrient cycling and the decomposition of complex organic materials introduced through organic amendments [89]. The presence of Gemmataceae, a member of the Planctomycetes group, also suggests an active microbial community involved in the degradation of complex polysaccharides and other recalcitrant organic compounds [90]. These metabolic functions are particularly significant in organically fertilized systems, where organic substrates supply diverse carbon sources that stimulate microbial activity and may facilitate soil biochemical processes [90].

In contrast, soils receiving chemical fertilizer showed higher abundances of Isosphaeraceae, Gemmatimonadaceae, and *Pirellula*, along with a modest increase in *Bradyrhizobium*. Members of the Planctomycetes group, including Isosphaeraceae and *Pirellula*, are known for their roles in the degradation of complex organic compounds and participation in nitrogen and carbon cycling processes [90]. Their presence indicates that these soils still harbor microbial communities capable of transforming nutrients. Gemmatimonadaceae, another taxon enriched in chemically fertilized soils, is known for its ability to accumulate polyphosphate and tolerate drought, suggesting potential adaptation to nutrient-enriched or resource-limited environments [91]. As *Bradyrhizobium* is an important nitrogen-fixing symbiont of legumes, its relatively higher abundance in chemically fertilized soils may reflect differences in microbial community dynamics and nitrogen availability between fertilization systems [86, 91].

Functional profiling predicted that the bacterial community may be influenced by the fertilization regime. Organic fertilizer amended soils showed relatively higher representation of functions associated with organic matter degradation, including xylan and chitin degradation, as well as a moderate representation of streptomycin-producing functions. This pattern may reflect differences in microbial processes related to nutrient turnover and overall soil health [92]. In contrast, chemically fertilized soils showed slightly higher representation of nitrogen-fixing and redox-associated functions. These patterns indicate differences in the relative representation of predicted functional groups between the two fertilization regimes, with organic fertilization showing comparatively greater representation of functions related to organic matter degradation and nutrient transformation [93].

Correlation analyses showed strong associations between soil nutrient concentrations and specific bacterial taxa, underscoring the possible influence of nutrient availability on microbial community structure. The observed association between P and taxa such as *Bradyrhizobium* suggests that P availability may be related to variations in microbial community structure, particularly in soils undergoing active biogeochemical cycling. However, these associations do not necessarily indicate a direct causal effect of P on the abundance or activity of these taxa [94]. In addition to nitrogen-fixation, several *Bradyrhizobium* species possess genes for P solubilization and uptake, such as alkaline phosphatases and high-affinity phosphate transport systems [95]. The strong association between P and *Bradyrhizobium* suggested that sufficient phosphorus supports nitrogen fixation and may facilitate microbial mechanisms for phosphorus mobilization. This interaction between phosphorus and nitrogen cycles may improve nutrient-use efficiency and supports long-term soil fertility [96]. Other nutrients, including S, K, and N, were strongly associated with bacterial taxa such as *Bacillus*, *Tumebacillus*, *Paenibacillus*, and Xanthobacteraceae*.* These results suggest that these nutrients may facilitate the proliferation of functionally important microbes involved in nutrient cycling and organic matter transformation [97, 98]. The close relationship between soil nutrient status and microbial community dynamics emphasizes the role of specific nutrients in shaping functionally relevant taxa. However, strong positive correlations between soil organic matter and organic carbon and key bacterial taxa, including *Bacillus*, Gemmataceae, *Tumebacillus*, *Paenibacillus*, and Xanthobacteraceae, underscore the critical role of organic matter in supporting microbial growth and activity [98]. However, a lower abundance of beneficial PGPB and organic matter-degrading microorganisms suggests that continual chemical fertilizer application may reduce microbial diversity and functional resilience, potentially jeopardizing long-term soil quality and ecosystem stability [99]. The results demonstrate that soil nutrient availability is a critical factor in shaping microbial communities by selectively enriching functionally important taxa responsible for key biogeochemical processes. Long-term use of organic fertilizer facilitates gradual nutrient release, which fosters a favorable environment for a broader spectrum of beneficial microorganisms. The predicted enrichment of potentially beneficial microbial taxa under organic fertilization may be associated with greater representation of microbial functional capacities related to nutrient cycling and soil fertility, although these predictions require experimental validation.

## Conclusion

In the present study, the distinct bacterial profiles observed between the two fertilization regimes indicate that long-term organic fertilization practice may promote a PGPB microbial community, including *Bacillus*, *Paenibacillus*, and *Tumebacillus*, which are more closely associated with plant growth promotion and active nutrient cycling, while prolong use of chemical fertilizer may facilitate microbial growth responsible for nutrient transformation, where the dominant taxa are mainly adapted to nutrient-enriched environments and have a limited range of plant-beneficial functions. Strong correlations between nutrient availability and functionally significant bacterial groups indicate that nutrient-rich environments enhance microbial activity, particularly when supplemented with organic amendments. The findings suggest that chemical and organic fertilization can differentially influence bacterial community structure, indicating that fertilizer management practices may act as important drivers of soil microbial community composition. The representation of potentially beneficial microbial taxa under organic fertilization may be associated with enhanced nutrient cycling and soil fertility, although these potential functions require further validation to establish their contribution to long-term agricultural sustainability. However, this preliminary study provides limited information on fertilizer composition, cropping history, or climatic variability. While plot management was consistent, the exclusion of these factors may constrain the interpretation of microbial dynamics. Although functional predictions were used to support the interpretation of the observed microbial community patterns, these predictions were derived from 16S rRNA gene sequencing data and were not experimentally validated. Future research that incorporates these factors will be required to achieve a more comprehensive understanding of the effects of fertilization on soil bacteriomes.

## Supplementary Information

Additional file 1: Table S1 Study sample information, SRA accession numbers of the 16S rRNA amplicon sequences mapped against bacterial taxa.

Additional file 2: Data S1 Physicochemical and heavy metal properties of soil samples.

Additional file 3: Data S2 Bacterial diversity analysis of the soil samples at phylum and genus level.

Additional file 4: Data S3 Relative abundance of bacterial community categories by their functional profiles.

## Data Availability

All sequencing data generated in this study have been deposited in the NCBI BioProject data-base under accession number PRJNA1415714, and are publicly available through the Sequence Read Archive (SRA) (SRR37017177, SRR37017176, SRR37017173, SRR37017183, SRR37017182, SRR37017181, SRR37017180, SRR37017179, SRR37017178, SRR37017174, SRR37017184, and SRR37017175).
