# Supplementary material for: Impact of extended fertilization on soil microbial diversity: a comparative full-length 16S rRNA amplicon analysis
Source: Genomics Inform. 2026 Oct 13;24:20. doi: 10.1186/s44342-026-00078-3 (PMC13660274; doi:10.1186/s44342-026-00078-3)
Supplement: Supplementary file 1 [file 44342_2026_78_MOESM1_ESM.docx]

**Table S1.** Study sample information, SRA accession numbers of the 16S rRNA amplicon sequences mapped against bacterial taxa.

| Sample ID | Sample title | Collection site | Coordinate | Category | Source | No. of reads (single-end) | GC% | SRA accessions |
| --- | --- | --- | --- | --- | --- | --- | --- | --- |
| CF_1 | Soil with chemical fertilizer treatment 1 | Bangladesh:Chattogram | 22.4716° N, 91.7877° E | Chemical fertilizer | Soil | 48,176 | 57 | SRR37017184 |
| CF_2 | Soil with chemical fertilizer treatment 2 | Bangladesh:Chattogram | 22.4716° N, 91.7877° E | Chemical fertilizer | Soil | 29,198 | 57 | SRR37017183 |
| CF_3 | Soil with chemical fertilizer treatment 3 | Bangladesh:Chattogram | 22.4716° N, 91.7877° E | Chemical fertilizer | Soil | 37,508 | 57 | SRR37017180 |
| CF_4 | Soil with chemical fertilizer treatment 4 | Bangladesh:Chattogram | 22.4716° N, 91.7877° E | Chemical fertilizer | Soil | 37,529 | 57 | SRR37017179 |
| CF_5 | Soil with chemical fertilizer treatment 5 | Bangladesh:Chattogram | 22.4716° N, 91.7877° E | Chemical fertilizer | Soil | 45,527 | 57 | SRR37017178 |
| CF_6 | Soil with chemical fertilizer treatment 6 | Bangladesh:Chattogram | 22.4716° N, 91.7877° E | Chemical fertilizer | Soil | 62,795 | 57 | SRR37017177 |
| OF_1 | Soil with organic fertilizer treatment 1 | Bangladesh:Chattogram | 22.4716° N, 91.7877° E | Organic fertilizer | Soil | 57,782 | 57 | SRR37017176 |
| OF_2 | Soil with organic fertilizer treatment 2 | Bangladesh:Chattogram | 22.4716° N, 91.7877° E | Organic fertilizer | Soil | 21,733 | 57 | SRR37017175 |
| OF_3 | Soil with organic fertilizer treatment 3 | Bangladesh:Chattogram | 22.4716° N, 91.7877° E | Organic fertilizer | Soil | 52,089 | 57 | SRR37017174 |
| OF_4 | Soil with organic fertilizer treatment 4 | Bangladesh:Chattogram | 22.4716° N, 91.7877° E | Organic fertilizer | Soil | 32,521 | 57 | SRR37017173 |
| OF_5 | Soil with organic fertilizer treatment 5 | Bangladesh:Chattogram | 22.4716° N, 91.7877° E | Organic fertilizer | Soil | 34,990 | 57 | SRR37017182 |
| OF_6 | Soil with organic fertilizer treatment 6 | Bangladesh:Chattogram | 22.4716° N, 91.7877° E | Organic fertilizer | Soil | 44,042 | 57 | SRR37017181 |
